# Locally adapted rhizobia strains for Sahelian nutritional security

**DOI:** 10.1093/sumbio/qvaf023

**Published:** 2025-09-23

**Authors:** Katherine Pitts Morgan, Amira Hachana, Carina Yiu, Amir Souissi, Lewis Ziska

**Affiliations:** New York University, Department of Environmental Studies, New York City, NY 10003, USA; National Institute of Agricultural Research of Tunisia, Laboratory of Agricultural Sciences and Techniques, Carthage University, Tunis 1004, Tunisia; Columbia University Mailman School of Public Health, Department of Environmental Health Sciences, New York City, NY 10032, USA; Swift Current Research and Development Centre, Agriculture and Agri-Food Canada, Swift Current, Saskatchewan, S9H 0K2, Canada; Columbia University Mailman School of Public Health, Department of Environmental Health Sciences, New York City, NY 10032, USA

## Abstract

Soil degradation and nitrogen depletion pose significant challenges to sustainable agricultural productivity and nutrition security in the Sahel region of Africa. While commercial rhizobial inoculants have been utilized as biofertilizers for leguminous crops, their effectiveness can be limited by poor adaptation to local conditions. Here, we call attention to the opportunity of locally adapted rhizobial inoculants to contribute to sustainable agriculture and nutrition security in the Sahel Region. Certain indigenous rhizobial strains across the African continent have demonstrated superior performance in nodulation, legume crop yields, and/or resilience to abiotic stresses compared to commercial inoculants. We propose a comprehensive framework that emphasizes (1) the selection of indigenous strains optimized for nitrogen fixation and abiotic stress tolerance, (2) matching inoculants with regionally important and underutilized legumes, and (3) ethical and broader considerations for developing inoculant formulations to enhance field performance. We stress that locally adapted rhizobial strains can contribute to enhanced nutrition security through improved legume crop yields, improve climate resilience, and potentially promote agricultural sustainability through reduced reliance on synthetic fertilizer inputs in the Sahel, with potential applications for other nitrogen-deficient regions globally. However, to be sustainable, this approach requires community-based participatory research, supportive policy frameworks, and investment in local capacity building.

**Sustainability Statement**

Through leveraging the untapped potential of indigenous rhizobia for nitrogen-deficient regions of the Sahel, the solutions proposed in this paper directly address nutrition security and sustainable agriculture (SDG 2), specifically advocating for the mitigation of protein deficiencies (Targets 2.1, 2.2), increasing small-scale production (Target 2.3), growing resilient agricultural practices (Target 2.4), and funding locally-tailored research and development (Target 2.6). These solutions are deeply integral in strengthening resilience and adaptive capacity to climate related disasters (SDG 13 [Climate Action], Target 13.1), catalyzing the restoration of degraded lands through targeted soil-microbiome revitalization (SDG 15 [Life on Land], Target 15.3), and promoting access to rhizobial population genetic resources and fair sharing of the benefits (Target 15.6).

## Introduction

**Figure 1.**
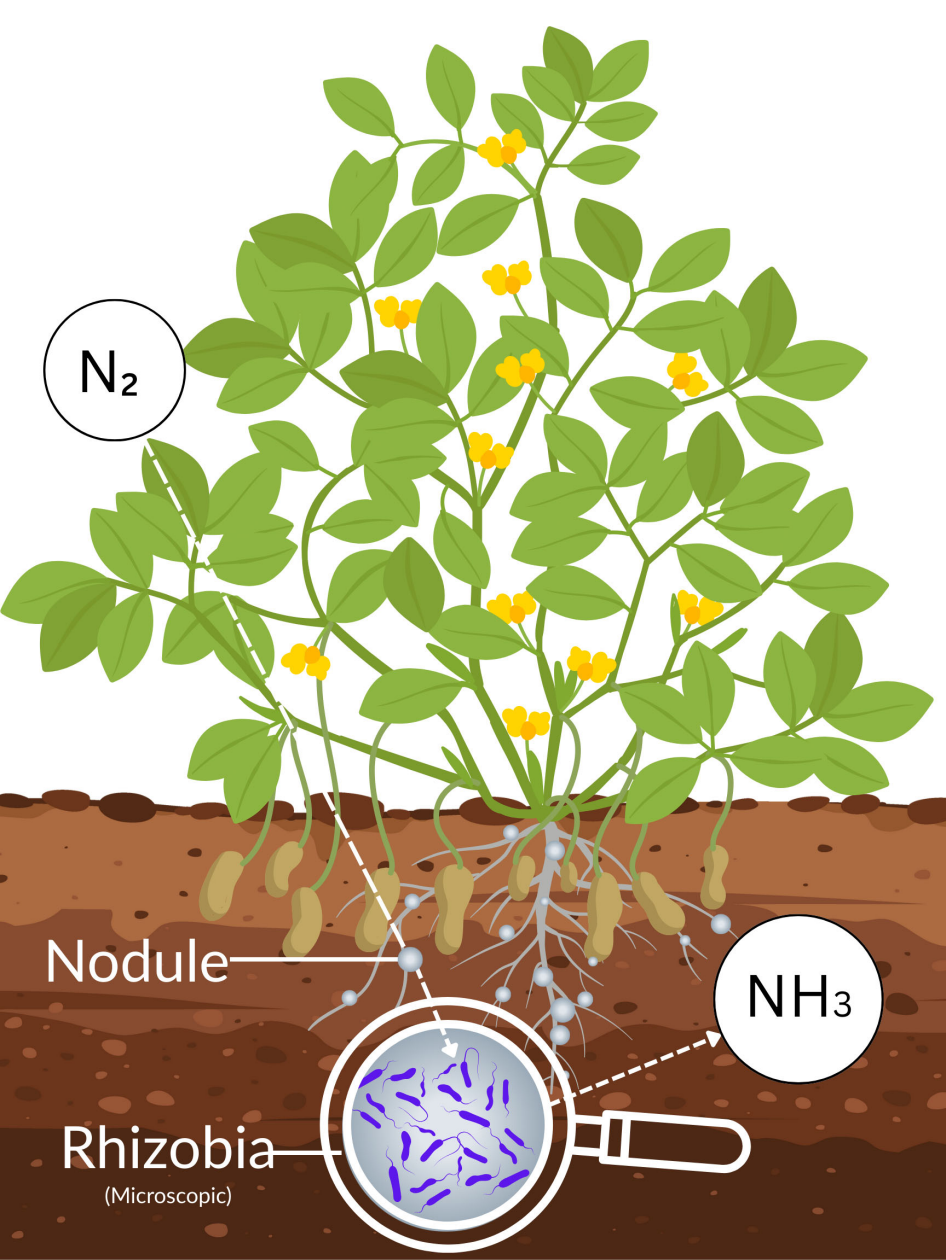
Illustrative Diagram Representing Symbiotic Nitrogen Fixation in Legume Root Nodules.

A range of soil microbes have coevolved alongside crop species, which can support growth, drought adaptation, or other functions. Rhizobia soil bacteria are a cornerstone example of such soil microbes due to their symbiotic relationship with legumes to facilitate biological nitrogen fixation (BNF), making them a potentially transformative, ecologically adapted, and locally relevant biofertilizer alternative to supplement or reduce reliance on synthetic fertilizers (Nziguheba et al. 2016, Dimkpa et al. 2023). Rhizobia bacteria show promise for climate change-resilient agriculture. Fig. 1 is an illustrative diagram representing the mutualistic relationship between a legume plant (depicted as *Arachis hypogaea*) and rhizobia. Atmospheric nitrogen (N₂) is absorbed into the soil and taken up within specialized structures on the legume's roots called nodules. Rhizobia reside within the nodules and produce nitrogenase, which converts the N₂ into ammonia (NH₃) to fuel plant growth (Kumar et al. 2021). Locally adapted rhizobia bacteria in the Sahel region can be used in inoculant biofertilizer formulations to create a sustainable and cost-effective solution that promotes legume growth and resilience and agroecosystem functioning. Such progress could ensure nitrogen security, a concept central to sustainable agriculture, by providing sufficient, and importantly, sustainable nitrogen in forms that support healthy crop growth.

Selection on crop traits relative to the environment has been a cornerstone of agricultural adaptation, but direct selection or inoculation of microbes in crop rhizospheres to match local conditions is a relatively recent development. This link between the rhizosphere, the zone where soil microorganisms interact with root-soil particles, and a complex ecosystem of fungi, bacteria, arthropods, nematodes, and other organisms (Hinsinger et al., 2009) may be fundamental in maintaining food systems where conventional Green Revolution agriculture has underperformed, and where the worst effects of climate change will impact nutrition security.

Centering the rhizosphere within agricultural research applications may be especially important in the Sahelian region, a unique transition zone in Africa between the more humid, semi-tropical savannah and the drier Saharan desert to the north, spanning a dozen countries, and home to over 150 million people (Dieng 2021). The Sahel region is challenged by degraded soils, low organic matter, phosphorus deficiencies, and nitrogen depletion, exacerbated by over-reliance on unsustainable farming practices from the Green Revolution (Moseley et al. 2010, Tully et al. 2015, Nziguheba et al. 2016). The soils, among the oldest and most weathered globally, are predominantly sandy with low cation exchange capacity, making them prone to nutrient leaching and poor fertility (Bationo et al. 2011; Valentin, 1994). In addition, poor infrastructure and transport can make access to fertilizer prohibitive (Breman et al. 2001). These challenges are compounded by frequent droughts, salinization, and extreme temperatures, all of which limit crop productivity and reduce the efficacy of conventional fertilizers (Nziguheba et al. 2016, Dimkpa et al. 2023). Further exacerbating the problem, the Sahelian region is experiencing climate change at rates faster than the global average. In the next 10–20 years, unprecedented changes in temperatures and precipitation are projected (Cepero et al. 2021).

Cowpea (*Vigna unguiculata*) and groundnut (*Arachis hypogaea*) are culturally relevant leguminous cornerstones of nutrition security in the Sahel, providing essential protein and micronutrients (Bakoye et al. 2019). Additionally, Nitrogen-fixing legumes, including the common bean, pea, and fava bean, often demonstrate better drought adaptation than plants reliant on external nitrogen sources due to their symbiosis with rhizobia (Antolín et al. 1995, Frechilla et al. 2000, Lodeiro et al. 2000, Staudinger et al. 2016, Amine-Khodja et al. 2022). Legumes enhance soil fertility and grow in relatively nitrogen-poor soils where they can also contribute to soil organic matter. As such, legume crops represent a dual potential for climate mitigation through increased carbon sequestration in infertile soils as well as reducing fossil energy inputs into the system by reducing nitrogen fertilizer needs (Monteoliva et al. 2023). From a climate-change standpoint, there is a clear imperative to advance our understanding of the legume-rhizobia symbiosis at the heart of this BNF. Investigating the rhizospheric microbiome associated with leguminous crops in the Sahel could potentially contribute to culturally relevant nutrition security, enhance soil health, and build soil carbon stocks. Crucial to this effort is evaluating ways and means to improve BNF within leguminous African crops in an uncertain climate.

While farmers have relied on microbes already present in the soil, there is increasing interest in the inoculation of soil with new rhizobial strains and rhizobial communities within biofertilizer formulations that promote plant growth by increasing nutrient availability or supply (Kumar et al. 2021). The use of rhizobial inoculants in West African and Sahelian agriculture systems has generally increased, although adoption is not yet widespread, common practice. A significant challenge arises with the use of these inoculants, as they are rarely tailored to local conditions. At present, when inoculants are applied to legumes in the Sahel region, they often use commercial rhizobial inoculants. While these inoculants can enhance BNF in some conditions, the symbiosis between a commercial rhizobial strain and a legume species can be soil-dependent– when interacting in sub-optimal rhizospheric conditions, this may lead to less efficient BNF. A number of studies specific to Africa have shown significant improvement in BNF in leguminous crops (cowpea, peanut, Bambara bean) fixation when indigenous rhizobial strains were used (Koskey et al. 2017, Wilson et al. 2021, Sene et al. 2023, Abdullahi and Abdullahi 2024).

We would argue that there is an immediate need to consider a regional shift toward tailored rhizobial inoculants—inoculants specifically designed using locally adapted rhizobia strains. Currently, researchers are investigating local strains of microbes associated with indigenous legume crops in Africa, which have been generically replaced in many areas, but have the potential to be revitalized for climate-resilience and/or nutrition, including grass pea, Kersting's groundnut, yam bean, and green grams (Paliwal et al. 2020). Promisingly, local rhizobia strains that nodulate cowpeas have shown resilience under drought conditions, offering a viable pathway for stabilizing yields in dry environments (Nziguheba et al. 2016).

**Figure 2.**
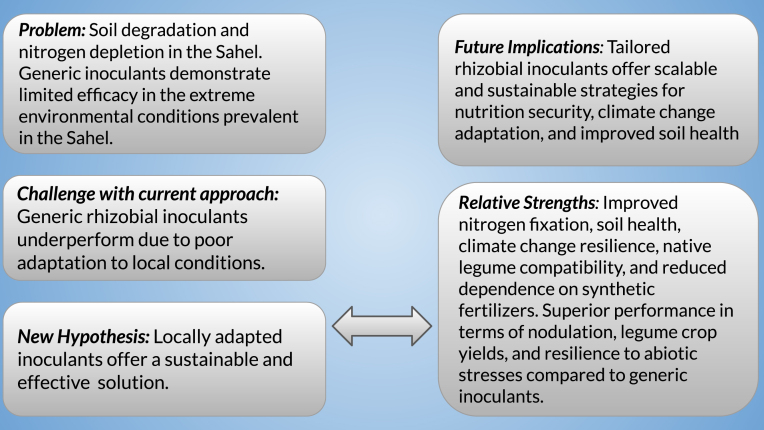
Framework for Developing Locally Adapted Rhizobia Inoculants in the Sahel.

Our objective is simple: to advocate for a shift from “one-size-fits-all” rhizobia inoculants toward a regional framework. We strongly encourage further investigation into the potential of locally tailored rhizobial inoculants in the Sahel to enhance soil health and food security. This requires centering local farmers and academic institutions in the characterization of the rhizospheric microbiome and investigating applications of locally adapted rhizobia with underutilized legumes and innovative inoculant formulations. The Framework for Developing Locally Adapted Rhizobial Inoculants in the Sahel (Fig. 2) aligns BNF strategies with the Sahel's agro-ecological and socio-economic realities. This approach can reduce dependency on synthetic fertilizers, restore degraded soils, and bolster nutrition security.

Symbioses between plants and soil microbes primarily involve the exchange of nutrients and water. A microbiome, the community of microorganisms within a given soil biome, can contain rhizobia- diazotrophic bacteria that form a symbiotic relationship with leguminous plants. Rhizobia stimulate plants through nod factor signaling to form root nodules that harbor the rhizobia, receiving energy via photosynthesis while reducing atmospheric nitrogen (N₂) into ammonia (NH₃), a plant-accessible form of nitrogen (van Heerwaarden et al. 2018, Virk et al. 2022). Mycorrhizal fungi (MF) are another key player in the rhizosphere. They form symbiotic associations with plant roots and share water and nutrients with the plants (Hrynkiewicz and Baum 2012, Singh et al. 2018, Alimi et al. 2021). While this perspective piece focuses solely on rhizobia, the combined action of rhizobia and MF can improve legume growth, yield, and resilience to environmental stresses like drought.

Beyond BNF, the rhizobia-legume symbiotic relationship provides several additional benefits to the legume partner, including enhanced nutrient uptake, phytohormone synthesis, and the release of enzymes that help plants cope with various environmental stresses (Olanrewaju et al. 2017, Patil et al. 2017, Fahde et al. 2023; Shahid et al., 2023; Zope et al., 2019). Once the symbiosis is established, legumes can better adapt to environmental stresses through changes in gene expression and epigenetic modifications, helping the plant manage stress more effectively (Zhang et al. 2024). For instance, in response to specific stresses like drought, rhizobial signaling enhances plant resilience by promoting stress-responsive pathways (Wekesa et al. 2022). Additionally, bacterial synthesis of 1-Aminocyclopropane-1-carboxylate (ACC) deaminase in the rhizosphere can reduce oxidative damage caused by ethylene accumulation, providing further protection to the plant under stress (del Carmen Orozco-Mosqueda et. al, 2020). They also synthesize siderophores, compounds that help mobilize iron and produce phytohormones like indole-3-acetic acid (IAA) to ultimately stimulate root growth under stress conditions (Lebrazi and Fikri-Benbrahim 2018, Hou et al. 2020). Furthermore, rhizobia has demonstrated the ability to mitigate a range of environmental stressors, including salinity, heavy metal toxicity, and drought, through mechanisms like biofilm formation, exopolysaccharide production, and chelation of harmful compounds (Kibret et al., 2024). These mechanisms help improve plant resilience and support sustainable crop production in degraded soils (Patil et al. 2017, Goyal and Habtewold 2023). In addition to fixing nitrogen, rhizobia can further improve plant nutrition by solubilizing phosphorus (Nziguheba et al. 2016, Dimkpa et al. 2023). This critical nutrient is often bound and unavailable in Sahelian soils due to their low pH and high phosphorus-fixing capacity (Oloo 2016). Rhizobia produce organic acids and phosphatases that release phosphorus from bound forms, thereby increasing its availability to plants in the rhizosphere (Nziguheba et al. 2016, Dimkpa et al. 2023).

## Materials and methods

The methodological approach for our perspective piece was informed by guidelines described by Ladle (2021), and the development involved a systematic and iterative process of literature review, synthesis, and conceptual framework construction. We conducted a broad review of existing transdisciplinary scientific literature across key domains, including microbiology, botany, ecology, agronomy, and sustainable agriculture, and relied on co-author regional expertise to further aggregate relevant publications. Relevant studies were sourced from diverse scholarly journals, white papers, and historical archives. Key research themes included the biophysics and biogeochemistry of the rhizosphere, distribution and characterization of rhizobia strains in Africa, and specifically the Sahel, climate change effects on food production, fertilizer supply chain vulnerabilities, and cutting-edge publications in legume-rhizobia symbiosis.

The gathered information was organized thematically in an organizational knowledge framework that allowed for a logical progression of arguments, from problem identification to proposed solutions and future research directions. Central to our methodology was the construction of a novel conceptual framework (represented visually in Fig. 2) that illustrates the problem-solution narrative of our perspective. This involved identifying the core issues of soil degradation and nitrogen depletion, analyzing the limitations of existing approaches (e.g. generic inoculants), and proposing a new hypothesis focused on locally adapted rhizobia strains.

## Results

### Recent innovations and advancing research in legume-rhizobia symbiosis

We posit that rhizobia research is a key pathway with transformative potential in addressing soil fertility and crop productivity, particularly in the Sahel. Over recent years, scholarly efforts to optimize indigenous rhizobia's BNF efficiency, improve their tolerance to abiotic stresses, and enhance plant growth-promoting properties have gained momentum (Sene et al. 2023). However, there is heavy reliance on commercial inoculants which has likely limited the benefits, as introduced formulations of strains frequently fail to align with local agro-ecological conditions and lead to inconsistent field results (Checcucci et al. 2017, Onishchuk et al. 2017). Recent advances, including the identification of indigenous strains capable of fixing substantial nitrogen levels, offer promising pathways to reduce dependence on synthetic fertilizers while promoting sustainable agricultural practices (Shina 2018, Abdullahi et al. 2020).

Another emerging research area sheds light on epigenetic mechanisms that govern rhizobia's symbiotic efficiency and adaptability to environmental stresses. Genome-wide analyses have revealed changes in DNA methylation during the transition between free-living and symbiotic states in species like *Bradyrhizobium diazoefficiens*. These changes moderate interactions between key genes involved in nodulation, BNF, and stress responses (Davis-Richardson et al. 2016). Interestingly, epigenetic DNA methylation patterns differ between free-living rhizobia and bacteroids within legume nodules in field pea (*Pisum sativum L.)* (Afonin et al., 2021).

Research has shown that DNA methylation of particular motifs in the rhizobial genome controls the expression of nodulation, BNF, and stress response genes, thus demonstrating the dynamic relationship between genetic and epigenetic factors that determine rhizobia's functionality, and revealing actively evolving research avenues (Ramsay et al. 2022). The complex epigenetic mechanisms that regulate rhizobia's symbiotic efficiency and environmental adaptability are only just beginning to be unraveled, and the insights that have been gained highlight broader regulatory processes, such as quorum sensing and horizontal gene transfer, that influence rhizobia adaptability and competitiveness. For example, quorum sensing allows bacteria to coordinate key physiological activities, including symbiosis, conjugation, antibiotic production, motility, sporulation, nutrient uptake, plant hormone production, and biofilm formation (Ramsay et al. 2022, Singh and Singh 2025). Together, quorum sensing and DNA methylation in rhizobia play a crucial role in regulating gene expression and behaviors that are essential for investigating rhizospheric interactions and their supposed effects on BNF (Ramsay et al. 2022).

The epigenetic priming for quorum sensing enables horizontal gene transfer, which leads to fast gene expression modifications and potentially enhances beneficial traits like better BNF efficiency and stress tolerance, according to Ramsay et al. (2022). The chemical signals between rhizobia and host plants result in increased nodule development while selecting more effective nitrogen-fixing symbiotic strains (Goyal et al. 2021). For example, the subpopulations of *Mesorhizobium* spp. undergo epigenetic priming for quorum sensing, which enables the horizontal transfer of integrative and conjugative elements that transform non-symbiotic *Mesorhizobium* spp. into nitrogen-fixing legume symbionts (Ramsay et al. 2022). Research needs include exploring the magnitude and predictability of these effects in field settings through detailed studies of symbiosis between different rhizobia and legume species.

### Challenges and opportunities: indigenous inoculants

Effective BNF in leguminous crop systems depends on rhizobial strains that are compatible with the target legume and local environmental conditions (Onishchuk et al. 2017). Unfortunately, non-locally adapted, lab-selected commercial inoculant strains have been shown in many cases to underperform in field conditions (Mendoza-Suárez et al. 2021, O'Callaghan et al. 2022, Malhotra et al. 2024). A recent study by Sene et al. (2023) assessed the inoculation efficiency of native and imported *Bradyrhizobium* strains for peanuts in Senegal: it showed that the response to inoculation was dependent on the cultivar, which points to the need for further response testing across different cultivars. Commercial strains and mixtures of strains can struggle to survive and persist in soils without adaptations to local abiotic stresses, undermining their ability to establish effective symbiosis with legume crops (Nziguheba et al. 2016, Kebede 2021). Key factors such as soil pH, temperature, salinity, moisture, and texture significantly influence rhizobial competitiveness and symbiotic efficiency, affecting the success of inoculants in agronomic applications (Saad et al. 2020; Vlassak et. al, 1997).

Concrete case examples can be found in the Sahel and across other African regions, where compelling quantitative data details improvements in yield, soil, and legume crop biochemical composition. In the sandy soils of north-eastern Egypt, Youseif et al. (2017) assessed seventeen native strains of faba bean-nodulating rhizobia, including *A. tumefaciens* (12) and *R. leguminosarum sv. viciae* (5), inoculating Faba bean *(Vicia faba L.)* in the field. In the first season, inoculation of field-grown faba bean showed significant improvements in seed yield (3.73–4.36 ton·ha − 1) and seed N-yield (138–153 Kg N·ha − 1), compared to the uninoculated control (48 kg N·ha − 1) that produced 2.97 Kg·ha − 1 and 95 kg N·ha − 1, respectively. In the second season, again, inoculation consistently improved seed yield and seed N-yield relative to the uninoculated control. While the benefits of improved yield are obvious, seed nitrogen (N) concentration can have a significant impact on seed protein content in plants, as N is a building block for protein. Such results infer the effects of the rhizosphere on human health and nutrition security based on the effects on seed N content and implications for meeting global protein demand. Specifically, the strain Rlv NGB-FR 126 demonstrated significant increments in seed yield (35%–48%) and seed N-yield (34%–49%) compared to the inorganic N fertilizers treatment (96 kg N·ha − 1) over both cropping seasons. Importantly, in this study, inoculation with native strains co-occurred with phosphate and potassium fertilization, which limits the full applicability of these findings to contexts with limited inorganic fertilizer inputs. Still, results demonstrate how faba bean inoculation with native rhizobial strains can reduce the need for inorganic N fertilization to achieve higher crop yield and seed N under low fertility soil conditions.

A study conducted in the Northern region of Ghana's Guinea savannah agroecological zone demonstrated the potential for the use of native elite *Bradyrhizobium* as cowpea and groundnut inoculants (Osei et al. 2020). At 8 of 12 field locations, cowpea cultivars inoculated with native elite *Bradyrhizobium* (KNUST 1002 and KNUST 1006) saw significant (P < 0.05) average yield increases of 63% (409 kg ha − 1) and 52% (339 kg ha − 1), respectively, when compared to a negative control. Groundnut cultivars exhibited similar positive responses, with 8 of 14 sites demonstrating significant (P < 0.05) average yield increases of 147 kg ha − 1 and 191 kg ha − 1 for populations inoculated with KNUST 1002 and KNUST 1006 strains, respectively. The overall findings of this study confirm that targeted *Bradyrhizobium* inoculation of cowpea and groundnut can lead to significant yield increases. However, the observed yield increases and inoculant efficacy varied across field locations, likely due to varying environmental conditions, related changes in host plant genotype-rhizobium strain compatibility, and local rhizobia population presence. Interestingly, KNUST 1002 inoculation offered the most significant yield increases in environments with relatively lower population levels of native rhizobium, while KNUST 1006 inoculation was more effective in environments with larger populations of native rhizobium. These findings offer insight into the complex relationships at play in the rhizosphere and highlight the need for further research to understand optimized use of indigenous inoculant strains; this will allow agrifood system actors to effectively select for inoculants based on local populations, soil composition, environmental conditions, and host plant genome.

In semi-arid regions of eastern Kenya, native and commercially available rhizobia were assessed to determine the effects on nodulation, growth, and yields of three local cowpea genotypes (Nyaga and Njeru 2020). Cowpea inoculated with the native isolates significantly (P < 0.05) increased yields by 22.7% (940.90 ± 71.88 kg ha − 1) when compared to the uninoculated control (766.60 ± 61.86 kg ha − 1) in the first season and a 28.6% increase in the second season. Importantly, the performance of rhizobia inoculants was influenced by season and the study location, denoting the importance of localized research to characterize superior native isolates with the potential to be developed into low-cost biofertilizer. For large-scale agricultural applications, inoculants well-adapted to the local soil conditions are necessary. The evidence indeed suggests the compelling opportunity of characterizing the soil microbiome and the implications of the rhizosphere in supporting human and planetary health (Nyaga and Njeru 2020).

The findings from these case studies present a strong case for a shift in how we approach agriculture from the purview of leveraging locally adapted microbial resources. For instance, a long-term study in Senegal revealed that a native shrub can surprisingly double millet yields and significantly enhance water efficiency during drought years (Bright et al. 2017). The long-term study in Senegal shows how a native leguminous shrub can double millet yields and dramatically increase water use efficiency during drought years, demonstrating a powerful mechanism for building climate resilience. Research from Egypt and Kenya demonstrates that native rhizobia can substantially improve crop yields and increase the nitrogen content of seeds, often surpassing the performance of commercial strains or synthetic fertilizers. Overall, these insights suggest that for sustainable agriculture in the Sahel to be effective, we need to rethink our strategies. It's essential to prioritize understanding and enhancing the diverse soil microbiomes in the region to build a sustainable and yield-secure agricultural future.

### Inoculants: research needs

Local rhizobial populations often exhibit high competitiveness in their environments, yet they are not always the most efficient at fixing nitrogen. This can lead to a “competition problem”, wherein the commercial, highly BNF-efficient rhizobia strains that are introduced into African soils are outcompeted and ineffective (Pastor-Bueis et al. 2019). In these cases, native strains dominate the root nodules but provide less nitrogen to the host plants (Triplett and Sadowsky 1992, Friesen 2012). However, even with non-native strains that are selected for BNF efficacy, the conditions under which they are developed do not reflect real-world scenarios. Notably, legume nodules can harbor multiple strains, allowing the plants to select multiple symbionts based on nutrient allocation while limiting resources to rhizobial underperformers, a process seemingly guided by the plant's ability to detect BNF levels (Mendoza-Suárez et al. 2021).

Another limitation of commercial rhizobial strains is their incompatibility with locally relevant legume species and underutilized varieties compared to more widespread legumes, such as the promiscuous soybean. Many commercial inoculants are developed for legumes grown in temperate regions. Such inoculants may fail to perform well with indigenous African legume species, such as Bambara groundnut, which dominate the Sahelian landscape. Finally, logistical and economic challenges for commercial inoculants need to be considered. These products often require specific storage conditions and transport infrastructure, and the cost of these inputs remains prohibitive for many smallholder farmers, especially if their performance is inconsistent and unreliable, failing to produce sufficient returns on investment (Wekesa et al. 2022, Mng'ong'o et al. 2023).

An increased focus on the isolation and characterization of indigenous rhizobial strains and the formulation and development of biofertilizers that perform well under Sahelian conditions may enhance their practicality and effectiveness (Mng'ong'o et al. 2023). Investments in local laboratory facilities and field trials are essential to address knowledge gaps in indigenous rhizobia diversity and distribution. Promising research frontiers include modifying strains to increase surface polysaccharides for better adherence to root hairs, optimizing nutrient acquisition systems, and engineering strains to produce antimicrobial bacteriosomes targeting inefficient soil strains (Mendoza-Suárez et al. 2021). However, prior to any transgenic or genome editing approaches, the characterization of locally adapted elite strains and screening for BNF effectiveness, as well as resilience to biotic and abiotic factors, should be the first step in Sahelian inoculant research. Future efforts should focus on isolating and characterizing high-performing indigenous strains for BNF and phosphorus solubilization, ensuring their compatibility with local legumes and resilience under Sahelian conditions (Checcucci et al. 2017, Mng'ong'o et al. 2023).

While transgenic or genome editing approaches hold promise, introducing genetically modified rhizobia into ecosystems still requires careful evaluation of potential ecological impacts, such as the disruption of local microbial communities, potentially leading to a decline in native rhizobial diversity and altering the dynamics of legume-rhizobia interactions (Taylor and Komatsu 2024). A precautionary approach to deploying selected inoculants should be pursued, accompanied by thorough ecological assessments before widespread application, including but not limited to checking the local diversity of rhizobia and performing experiments to see how the introduced strain could impact the local soil type and BNF efficacy.

Finally, it is essential to ensure that any research and development is conducted with rigorous ethics and the consent and oversight of farmers to collect local soil samples and participate in experimental trials. (Ntsomboh-Ntsefong et al. 2023). Renowned scholar and member of the Alliance for Food Sovereignty in Africa, Dr. Million Belay, asserts that “Technology could revolutionise Africa's agriculture if it is employed with the participation of farmers, and if it is used in a way that makes sense contextually. It must be designed with small-scale food producers in mind, allowing for scalability and adaptability to local settings” (Belay 2024). As such, any developments in locally tailored biofertilizers should be accessible and affordable for smallholders. Such innovations could enhance crop yields and reduce reliance on chemical fertilizers, but if co-opted by large agribusiness (as opposed to led through equitable Public-Private Partnerships or Non-Governmental Organizations and Farmer Organizations), it also risks exacerbating inequalities in access to technology and resources (Chibeba et al. 2017, Batstone et al. 2020).

Given the diverse knowledge presented above, we advocate for strain selection of locally adapted indigenous rhizobia as a primary and promising approach due to their inherent adaptation to challenging local conditions. The potential of transgenic approaches and genome editing to improve competitiveness and stress tolerance exists but their implementation requires thorough evaluation of ecological, ethical and regulatory factors in the Sahelian region. The following road map in Table 1 outlines the development process for locally adapted rhizobia strains, including strain selection, breeding, transgenic approaches, and genome editing.

**Table 1.** Approaches to developing locally adapted rhizobia strains for Sahelian nutrition security.

Approach	Description
Strain selection*	Strain Selection entails investigating elite Indigenous strains through significant research efforts involving isolation, characterization, and screening of locally adapted rhizobial strains to the unique soil types, abiotic conditions, and legume varieties of the Sahel. While these strains hold potential to be a low-cost and sustainable alternative to expensive synthetic fertilizers, even locally adapted strains might exhibit performance and competitive variability depending on specific soil properties, legume cultivars, and environmental fluctuations.
Selective breeding	Through careful selection and propagation of strains consistently showing high nitrogen fixation with specific local legumes, overall symbiotic efficiency over generations of selection might be enhanced. This approach can be time-consuming, requiring multiple cycles of selection and evaluation. Crosses or selections might lead to unpredictable combinations of traits, some of which could be undesirable.
Transgenic approaches	Transgenic approaches hold potential to enhance the competitiveness of native rhizobia through genetic modification of elite strains, such as through mechanisms to increase production of surface polysaccharides. However, more research is needed as these approaches raise ethical and ecological concerns that require careful consideration and significant regulatory oversight due to potential unintended consequences. The genetic instability of inoculant strains, including genetically modified ones, could transfer symbiotic plasmids to native, less effective rhizobia.
Genome editing	Genome editing techniques (e.g. CRISPR-Cas9) offer a more precise way to modify specific genes or regions in the rhizobial genome compared to traditional genetic modification. Genome editing tools can be used to precisely study the function of specific genes involved in symbiosis and adaptation to the Sahelian environment, aiding in the identification of key targets for improvement. While more precise, genome editing still involves genetic modification and may raise similar ecological and ethical concerns regarding impacts on the soil microbiome and unintended consequences.

## Discussion

The Sahel's unique agricultural and ecological challenges demand comprehensive, participatory, and scalable solutions to improve soil fertility, increase crop yields, and enhance nutrition security. This system-based approach will require ethical innovation in scientific research and development, policy frameworks, and farmer-centric capacity building.

### Scientific innovation

It is well established that there is an immediate need to undertake screening of locally adapted rhizobial strains to assess opportunities for improved efficacy of BNF for soil types, climatic conditions, and legume varieties indigenous to the Sahel (Onishchuk et al. 2017, Pastor-Bueis et al. 2019). Native rhizobia isolates could be screened for BNF efficiency, competitivity against other native rhizobia, and tolerance to abiotic stresses such as drought, salinity, and high temperatures (Checcucci et al. 2017, Wekesa et al. 2022). Such an approach has been essential in identifying performant indigenous rhizobia strains and can enhance nodulation and growth under drought conditions for cowpeas, for example (Nziguheba et al. 2016). Matching inoculants to regionally important legumes like cowpea, groundnut, and Bambara groundnut ensures successful nodulation and BNF, improving adaptability to climate variation. Underutilized legumes as “opportunity crops” should be prioritized, as overcoming symbiosis establishment barriers through human intervention via inoculation could lead to significant improvements in soil fertility and human nutrition (Mpepereki et al. 2000, Simonsen et al. 2017).

There is a need to determine the potential for inoculants that optimize the survival, persistence, and competitiveness in relation to other native soil microorganisms. Such optimization may include the application of protective coatings and the development of stress-tolerant microbial consortia (Kebede 2021, Mng'ong'o et al. 2023). Such efforts can help ensure the selection and application of rhizobial strains capable of thriving in the changing environmental conditions unique to the Sahel and may particularly be helpful for improving the efficiency of symbiotic BNF in Sahelian regions affected by high salt concentrations (Checcucci et al. 2017, Dimkpa et al. 2023). In addition, optimizing the delivery of rhizobia through innovative inoculant formulations may enhance field application. Research suggests that there may be benefits to replacing traditional solid rhizobial carriers with locally available materials like compost or biochar, which are more sustainable and accessible (Mendoza-Suárez et al. 2021).

### Policy frameworks for scaling soil transformation

Smallholder farmers in the Sahel face significant socioeconomic barriers, including limited access to fertilizers and high costs of agricultural inputs (Masso et al. 2015, Raimi et al. 2017). Political instability and climate change further amplify these vulnerabilities, making low-cost, nature-based solutions critical for enhancing the region's agricultural resilience and food security as the climate changes. Lower input costs, particularly in the face of rising fertilizer prices and environmental degradation, could be major natural incentives for farmer adoption of locally adapted rhizobial inoculant biofertilizers.

At the governmental level, there is a need for collaboration to develop regional frameworks to standardize participatory research methods to characterize selected indigenous Sahelian rhizobia, as well as consider quality control and distribution of rhizobia inoculants. Sahelian governments could prioritize policies that support community-based research that engages smallholder farmers regarding the production and distribution of tailored rhizobia inoculants (Bala et al. 2011, Wekesa et al. 2022). Cross-border initiatives can help scale innovations while fostering knowledge-sharing and capacity-building (Bala et al. 2011, Nziguheba et al. 2016). Finally, national agricultural strategies should also integrate rhizobia-based solutions into broader climate-adaptation plans, leveraging their ability to enhance soil fertility while reducing greenhouse gas emissions (Krause et al. 2022).

Farmers and researchers should perform participatory research to identify high-performing native rhizobia in local soils that show effective nitrogen-fixing abilities and symbiotic relationships with local legume crops. Once elite regional strains are identified, they can be multiplied in low-cost biofertilizer production hubs using liquid or peat-based carriers to ensure viability and field effectiveness. The local production hubs could enhance the availability of inoculants in rural areas and integrate with efficient supply chains to distribute inoculants, ensuring they are affordable for farmers. Supply chain developments related to these efforts should be developed in a way that minimizes plastic and other waste, accompanied by innovative recycling of collected plastics to ensure smaller adverse environmental impacts. Finally, effective technology adoption requires farmer education and capacity-building programs to bridge knowledge gaps about rhizobia inoculants and provide training on inoculant application techniques and integration with existing cropping systems (Mpepereki et al. 2000, Pastor-Bueis et al. 2019).

Scaling the application of rhizobia-based biofertilizers necessitates improved rural infrastructure development for storage facilities, transportation systems, and local production centers. The availability of inoculants in rural areas where commercial fertilizers are scarce or cost-prohibitive will be important for the successful adoption/uptake of inoculants throughout smallholder farming systems based on resilient distribution networks that reach remote areas (Raimi et al. 2017, Wekesa et al. 2022). Public-private partnerships should address smallholder farmers' logistical challenges by providing funding and partnership support for knowledge transfer and capacity-building initiatives. Such proposed strategies for Sahelian soil transformation hold intrinsic value but their practical implementation will be challenging because the region presents unique obstacles to overcome. Implementation strategies must be inherently adaptive and flexible, capable of responding to evolving climate conditions, emerging socio-economic realities, and continuous monitoring, evaluation, and a willingness to adjust approaches based on field observations and emerging scientific understanding.

### Next steps

Future efforts should encompass the evaluation of genotypes and the replication of indigenous rhizobia through experimental studies, with subsequent field trial evaluations of results aggregated across local legume varieties relative to climate vulnerability throughout the Sahelian region. Such an approach would include *in situ* evaluation of rhizobia relative to current and future leguminous crop planting and modeling of climate change and field trial data to determine agricultural management recommendations for farmers across the Sahel.

The research and application of rhizospheric studies requires joint efforts between countries and regions to achieve responsible agricultural development, which supports biodiversity conservation and sustainable use. The UN Conference of the Parties (COP) to the Convention on Biological Diversity (CBD) created the Kunming-Montreal Global Biodiversity Framework to protect biodiversity through sustainable use and benefit distribution while establishing essential implementation measures that defend local rights and prevent biopiracy. Section C recognizes local communities as vital entities that protect biodiversity, the rhizospheric microbiome, while participating in its preservation and sustainable uses. The implementation of policies should include provisions for fair genetic resource access and informed consent from farming communities and local resource protection to prevent commercial monopolization for achieving equitable, sustainable, and ethical benefits from elite Sahelian rhizobia.

Research and development efforts to harness the potential of elite rhizobia strains must proceed with caution, particularly regarding unintended consequences that may arise from manipulating these microorganisms. The long-term effects of genetically engineered rhizobia on the broader ecosystem are yet to be fully understood. Therefore, it is crucial that research and development of rhizobia inoculants be community-based, aligned with international biodiversity standards, and include rigorous testing in field conditions.

## Conclusion

This perspective provides a robust framework for the development of locally adapted rhizobia inoculants, which can be a key component for improving agricultural sustainability, soil health, and food security in the climate-vulnerable Sahel region. It seeks to underscore the potential of using native elite rhizobial inoculants in local settings, where strains are chosen and used to address the problems of particular soils, legume species, and climatic stresses (Checcucciet al. et al. 2017, Wekesa et al. 2022). By tailoring rhizobia inoculants to local conditions, the Sahel can serve as a model for other nitrogen-deficient regions seeking sustainable, low-cost alternatives to chemical fertilizers. Policy support, community engagement, and investment in research are needed to realize the full potential of this approach (Kebede 2021, Mng'ong'o et al. 2023).

In conclusion, advancing locally adapted rhizobia research and application presents a potentially transformative opportunity for addressing the intertwined challenges of food insecurity, soil degradation, and climate damage in the Sahel. As the Sahel faces escalating environmental and socio-economic pressures, exploration of localized soil microbiomes offers a path to long-term agricultural sustainability and human well-being in a rapidly changing climate.

## Data Availability

No new data were generated or analyzed in support of this research.
